# Corn Silk (*Zea mays L.*) Induced Apoptosis in Human Breast Cancer (MCF-7) Cells via the ROS-Mediated Mitochondrial Pathway

**DOI:** 10.1155/2019/9789241

**Published:** 2019-10-20

**Authors:** Mai M. Al-Oqail, Ebtesam S. Al-Sheddi, Nida N. Farshori, Shaza M. Al-Massarani, Eman A. Al-Turki, Javed Ahmad, Abdulaziz A. Al-Khedhairy, Maqsood A. Siddiqui

**Affiliations:** ^1^Department of Pharmacognosy, College of Pharmacy, King Saud University, Riyadh, Saudi Arabia; ^2^Al-Jeraisy Chair for DNA Research, Zoology Department, College of Science, King Saud University, P.O. Box 2455, Riyadh 11451, Saudi Arabia; ^3^Zoology Department, College of Science, King Saud University, P.O. Box 2455, Riyadh 11451, Saudi Arabia

## Abstract

Cancer has been recognized as one of the life-threating diseases. Breast cancer is a leading cause of mortality among women. In spite of current developments in the therapy and diagnosis of cancer, the survival rate is still less. Recently, plant-derived natural products gain attention as anticancer agents due to the nontoxic nature. Therefore, the aim of present study was to investigate the anticancer capacity of corn silk extract (CSE) on human breast cancer (MCF-7) and normal human mesenchymal (hMSC-TERT4) cells. Following 24 h treatment to corn silk extract, the cytotoxicity was assessed by MTT, NRU, and morphological assays. The oxidative stress markers (GSH and LPO), ROS production, MMP change, and expression of apoptotic marker genes (p53, Bax, Bcl-2, caspase-3, and caspase-9) were also studied in MCF-7 cells treated at 250 to 1000 *μ*g/ml of CSE for 24 h. Our results showed that CSE decreased the cell viability and increased the apoptosis in a dose-dependent manner. The level of LPO and ROS production was found significantly higher; however, GSH and MMP level was observed lower in CSE-treated MCF-7 cells. The real-time PCR data showed a significant upregulation in p53, Bax, caspase-3, and caspase-9 and downregulation in the mRNA expression of Bcl-2 genes in MCF-7 cells exposed to CSE. Collectively, the data from this study stated that corn silk extract induced apoptosis via the ROS-mediated mitochondrial pathway in MCF-7 cells.

## 1. Introduction

Breast cancer (BC) is the most frequently occurring cancer in females. Overall, BC is the second most common cancer with two million new cases in 2018 [1]. As per the data collected by the National Centre for Health statistics, 600920 demises and 1688780 fresh cancer cases were anticipated to arise in the women of United States in 2017 [2]. According to the report published by Jemal et al. [3], the worldwide incidence of breast cancer will keep increasing annually by 0.4%. Thus, the prevention of BC is a challenge between the scientists and searchers working in the area around the world. The increasing rate of BC generating economic burden to the society demands for the search of a novel, effective, and beneficial procedure. The available chemotherapeutic drugs, paclitaxel and anthracyclines, are known to constrain cancer growth and induce cancer cell apoptosis [4]. Nevertheless, these drugs are not sensitive to some of the patients and leads to the unwanted side effects to the healthy cells as well [5]. Hence, it is required to find a powerful, targeted, and nontoxic therapeutic agent to treat BC using natural products.

Plants as a natural product have received great attention as an anticancer agent with less side effects [6–8]. Corn silk (*Stigma maydis*), a byproduct of yellow or green maize (Zea mays L.), is well known for the effective treatment of nephritis, hypertension, prostatitis, and urinary tract infections [9]. In a traditional system of medicine, the corn silk has been used in several areas of the world including the United States, China, France, and Turkey [10]. The extracts of corn silk contain good quantity of a type of flavonoid, maysin, which is specific to corn [11, 12]. Maysin is a flavone glycoside encompassing luteolin, a biologically active agent known for its antioxidant and anticancer potential [13]. The high maysin corn silk extracts have been proven to be beneficial in reducing the body weight and fat deposition in C57BL/6J mice [14]. Bai et al. [15] have also shown the protective effects of ethanolic maize silk extract on radiation-induced oxidative stress. The administration of aqueous extract of corn silk at 100–400 mg/kg b.w. on hematological and lipid parameters in rats [16] and male ICR mice treated for 4 weeks at 500 mg/kg b.w. of corn silk extracts has been found nontoxic [10].

Our literature survey revealed that corn silk has extensively been reported to have substantial bioactivities such as antioxidant [17], antidiabetic [18], antibacterial [19], antifatigue [20], antidepressant [21], and antitumor [22] activities. The antiproliferative activity of corn silk extract against the LoVo (human colon cancer) cell line [23] and apoptosis in C6 rat glioma cells through mitochondrial ROS [24] have been reported. To the best of our knowledge, the mechanisms of anticancer effects of corn silk extract (CSE) against MCF-7 (human breast cancer) cells have not been studied yet. Hence, we aimed to present investigation firstly to assess the cytotoxic potential of CSE against MCF-7 and TERT4 cell lines and secondly to study the mechanism(s) of CSE inducing oxidative stress, ROS production, and apoptosis in the MCF-7 cell line.

## 2. Materials and Methods

### 2.1. Preparation of CSE

The fresh corn silk was obtained from the local market of Riyadh, Saudi Arabia. The corn silk was washed, air-dried, and powdered for further use. The methanolic extract was obtained by maceration. Briefly, 25 g of powdered corn silk was extracted with 100 ml methanol/water (80%, *v*/*v*). The filtrate was collected and dried in a rotary evaporator at 40°C till dryness. The dried extract was stored at 4°C for further use. The extract was diluted in DMSO, and the final concentration of DMSO for cytotoxicity assessment and other assays was 0.004%.

### 2.2. Cell Culture

Human breast cancer MCF-7 and normal cell line hMSC-TERT4 were obtained from ATCC. MCF-7 and hMSC-TERT4 cells were grown in DMEM and MEM, respectively, complemented with fetal bovine serum (10%) in a CO_2_ incubator (5% CO_2_, 95% air) at 37°C.

### 2.3. Cytotoxicity of Corn Silk Extract (CSE)

Cytotoxic effects of CSE were performed using MTT assay, NRU assay, and morphological assessment using the method established by us [25]. To assess the cytotoxic effects of CSE, the MCF-7 and TERT4 cells were seeded in a 96-well culture plate at a density of 10,000 cells per well. After overnight incubation, the cells were exposed to different concentrations (10-1000 *μ*g/ml) of CSE for 24 h using culture medium in untreated control. The percent cell viability was calculated using the formula below:

(1) $$\begin{array}{c} \%\text{cell viability}=\frac{\left(\text{mean absorbance of treatment}\right)}{\left(\text{mean absorbance of control}\right)}\times 100. \end{array}$$

For, morphological assessments, the MCF-7 and TERT4 cells were seeded in a 96-well culture plate at a density of 1 × 10^4^ cells/well. The cells exposed to different concentrations of CSE were analyzed under the phase contrast inverted microscope at 20x magnification power.

### 2.4. Effects of CSE on Oxidative Stress Markers and ROS Generation

The CSE inducing oxidative stress was evaluated by measuring the glutathione level, LPO, and ROS generation in the MCF-7 cell line.

#### 2.4.1. GSH Measurement

The total glutathione was measured according to Chandra et al.'s study [26]. Following the protocol, MCF-7 cells seeded in 6-well culture plates (1 × 10^5^) were left overnight in a CO_2_ incubator. Then, cells were exposed to 0, 250, 500, and 1000 *μ*g/ml of CSE for 24 h. After exposure, cells were harvested and protein was precipitated in 1 ml of 10% TCA by sonication. Then, the supernatant was collected by centrifugation and 2 ml (0.4 M Tris buffer, 0.02 M EDTA) was added to the supernatant. Subsequently, 0.01 M DTNB (5,5′-dithionitrobenzoic acid) was added and incubated for 10 minutes at 37°C. The absorbance of developed color was read at 550 nm wavelength.

#### 2.4.2. Lipid Peroxidation (LPO)

To measure the lipid peroxidation induced by CSE, MCF-7 cells (1 × 10^5^) were seeded in 6-well plates and exposed at 0, 250, 500, and 1000 *μ*g/ml of CSE for 24 h. The LPO in MCF-7 cells was measured by the TBARS (thiobarbituric acid reactive substances) method [27]. After 24 h treatment, MCF-7 cells were collected and sonicated in 1.15% KCl. Following centrifugation, 2 ml of the TBA reagent (15% TCA, 0.7% TBA, and 0.25 N HCl) was added to the supernatant. Then, the solution was boiled at 100°C for 15 minutes, and the absorbance of the supernatant was measured after centrifugation at 1000 rpm for 10 minutes.

#### 2.4.3. Measurement of ROS Production

Intercellular ROS production in MCF-7 cells was examined using the DCF-DA (2,7-dichloroflourescin diacetate) probe. DCF-DA is commonly used for the detection of ROS generation in cells, since this probe is cell permeable and can enter into the cells and react with ROS to form DCF (dichlorofluorescein), a fluorescent complex. The ROS generation was done using the protocol of Siddiqui et al. [28]. Following the protocol, MCF-7 cells were seeded in 24-well culture plates (2 × 10^4^ cells) and incubated in a CO_2_ incubator for overnight. The cells were exposed at 0, 250, 500, and 1000 *μ*g/ml of CSE for 24 h. After respective exposures, 20 *μ*M of dye was added to each well and further incubated for 1 hour in the dark. The fluorescence intensity of the DCF was analyzed under the fluorescence microscope. To measure the quantitative ROS generation, the fluorescence of the cells was measured at 485 nm excitation and 530 nm emission using a fluorescent reader.

### 2.5. Determination of MMP (*ΔΨ*m)

The MMP level in MCF-7 cells was analyzed using Rhodamine-123 fluorescence dye (Rh-123) [28]. As per protocol, MCF-7 cells seeded in 24-well culture plates (2 × 10^4^) were left overnight in a CO_2_ incubator. Following the exposure of CSE for 24 h at 250, 500, and 1000 *μ*g/ml, cells were washed with PBS. Then, cells were exposed to Rh-123 dye for 60 minutes in the dark at 37°C. The fluorescence intensity of the Rh-123 dye was observed under the fluorescence microscope, and the fluorescence intensity of Rh-123 in MCF-7 cells was also measured at 485 nm excitation and 530 nm emission wavelengths using a spectrofluorometer for quantitative analysis.

### 2.6. Analysis of Apoptotic Markers Genes

The real-time PCR (RT-PCR^q^) analysis was conducted to measure the mRNA expression of proapoptotic genes (p53, Bax, caspase-3, and caspase-9) and antiapoptotic gene (Bcl-2) in MCF-7 cells treated with CSE according to the method of Al-Oqail et al. [29]. Following the protocol, MCF-7 cells were harvested and seeded in 6-well culture plates (1 × 10^6^ cells) and allowed to adhere overnight. Cells were exposed to 0, 250, 500, and 1000 *μ*g/ml of corn seed extract for 24 h. Then, the total RNA was isolated from treated and untreated sets using an RNeasy mini kit (Qiagen). The integrity of RNA was checked using a gel documentation system on 1% gel. Further, cDNA was synthesized by reverse transcriptase using M-MLV and oligo (dT) primers (Promega). The RT-PCR^q^ was performed by a LightCycler® 480 instrument. The expression of apoptosis-related genes was normalized to a housekeeping gene, *β*-actin. The details of the primer sequences for p53, Bax, caspase-3, caspase-9, Bcl-2, and *β*-actin are reported in our earlier publication [29].

### 2.7. Statistical Analysis

The statistical analysis was done using one-way ANOVA and post hoc Dunnett's test to analyze the significant differences between the control and treated groups. The values showing *p* < 0.05 were considered statistically significant.

## 3. Results and Discussion

### 3.1. Cytotoxic Effects of CSE on MCF-7 and TERT4

**Figure 1:**
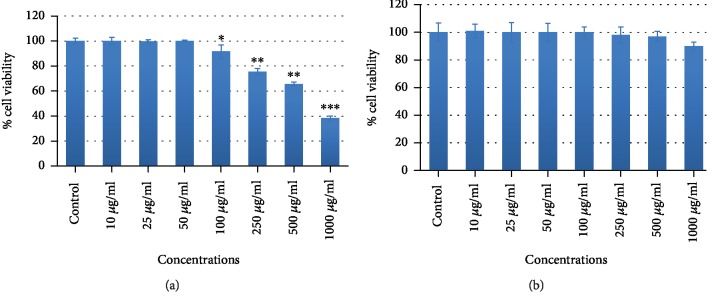
Cytotoxic effects of corn silk extract (CSE) by MTT assay on MCF-7 (a) and TERT4 cells (b). The cells were exposed to various concentrations of CSE for 24 h. Data are presented as the mean ± SD of three different experiments. ^∗^*p* < 0.05, ^∗∗^*p* < 0.01, and ^∗∗∗^*p* < 0.001 compared to control.

**Figure 2:**
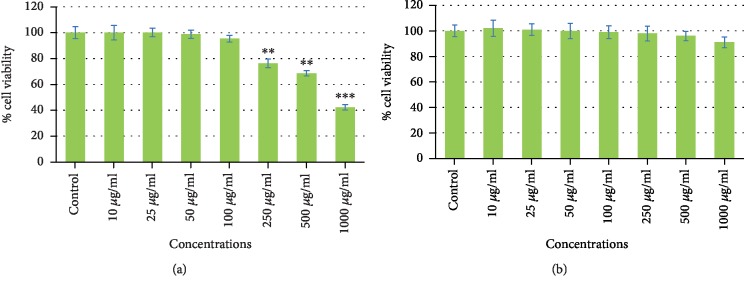
Cytotoxic effects of corn silk extract (CSE) by neutral red uptake assay on MCF-7 (a) and TERT4 cells (b). The cells were exposed to various concentrations of CSE for 24 h. Data are presented as the mean ± SD of three different experiments. ^∗^*p* < 0.05, ^∗∗^*p* < 0.01, and ^∗∗∗^*p* < 0.001 compared to control.

**Figure 3:**
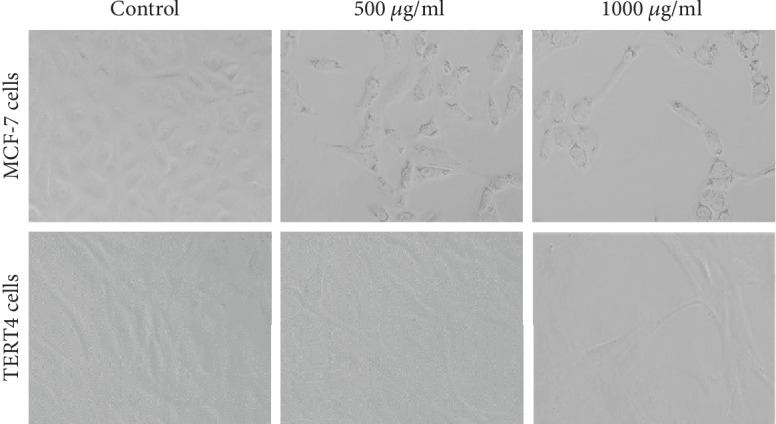
Representative images of morphological changes in MCF-7 and TERT4 cell lines. MCF-7 and TERT4 cells were exposed to different concentrations of corn silk extract (CSE) for 24 h. Images were grabbed using a phase contrast inverted microscope at 20x magnification power.

To assess the cytotoxic potential of CSE, MCF-7 and TERT4 cells were exposed to increasing concentrations (10, 25, 50, 100, 250, 500, and 1000 *μ*g/ml) of CSE for 24 h. The highlights of the cytotoxic results obtained by MTT assay, NRU assay, and morphological alterations are summarized in Figures 1–3, respectively. As shown in Figure 1, MTT assay revealed a concentration-dependent cytotoxicity in MCF-7 cell lines. A significant dose-dependent decrease in the cell viability of MCF-7 (Figure 1(a)) was found at 100 *μ*g/ml or higher concentrations of CSE exposed for 24 h whereas at the same concentration of CSE, TERT4 cell viability was not reduced (Figure 1(b)). Similar to MTT assay, the NRU assay also revealed a concentration-dependent cytotoxicity in MCF-7. 100 *μ*g/ml or higher concentrations of CSE were also found to decrease the viability of MCF-7 cells (Figure 2(a)) by NRU assay exposed for 24 h whereas the viability of TERT4 cells was not reduced at similar concentrations of CSE (Figure 2(b)). The viability of MCF-7 cells at 250, 500, and 1000 *μ*g/ml of CSE was found as 75%, 65%, and 38% by MTT and 76%, 68%, and 42% by NRU assays, respectively. The morphological alterations observed under the microscope in MCF-7 and TERT4 cells are presented in Figure 3. The CSE at 500 and 1000 *μ*g/ml reduced the number of MCF-7 cells, which become rounded and smaller in size. However, there was no significant effect in the morphology of TERT4 cells observed at tested concentrations. The MCF-7 cells were found cytotoxic to CSE; therefore, we further discovered the possible mechanism(s) of CSE inducing apoptosis in MCF-7 cells. In the present investigation, we used MTT and neutral red uptake assays to assess the cytotoxic potential of CSE because of their different mode of action. The MTT assay is mainly based on the conversion of MTT in the mitochondria [30]. The neutral red uptake assay is colorimetric assay measuring the uptake of the dye by functional lysosomes [31]. Both the assays (MTT and NRU) revealed a significant-dose dependent decrease in the viability of MCF-7 cells. However, NRU assay showed little less cytotoxicity as compared to MTT assay. It has been reported previously that different cytotoxicity assays can give different results depending upon the test material used and the cytotoxicity assay employed [32].

### 3.2. CSE Induced Oxidative Stress and ROS Generation in MCF-7 Cells

**Figure 4:**
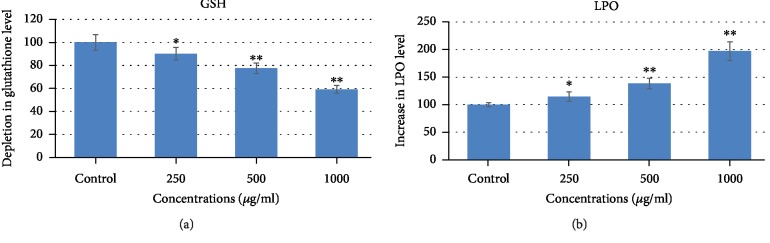
Effects of corn silk extract (CSE) on oxidative stress markers in MCF-7 cells. (a) Depletion in glutathione (GSH) level and (b) induction in lipid peroxidation (LPO). Data are presented as the mean ± SD of three different experiments. ^∗^*p* < 0.05 and ^∗∗^*p* < 0.01 and compared to control.

**Figure 5:**
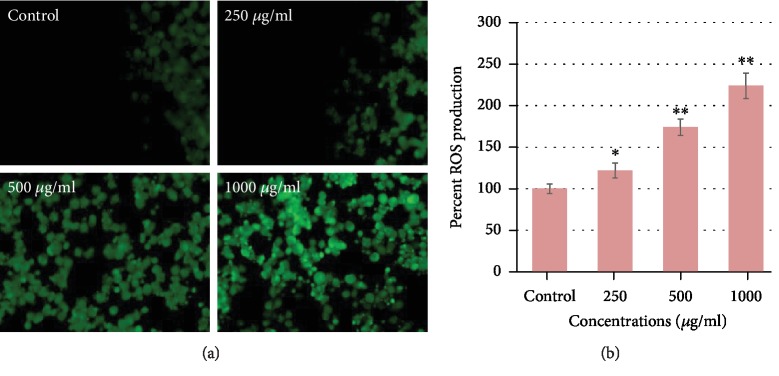
ROS generation induced by corn silk extract (CSE) in MCF-7 cells. (a) Fluorescence images showing intensity of DCF-DA dye after the CSE exposure at 0, 250, 500, and 1000 *μ*g/ml for 24 h. (b) The graph shows the percent ROS generation in MCF-7 cells. Data are presented as the mean ± SD of three different experiments. ^∗^*p* < 0.05 and ^∗∗^*p* < 0.01 and compared to control.

As presented in Figure 4(a), a depletion in the GSH level was observed after 24 h exposure of CSE and this consequence was found in a concentration-dependent manner. We observed that MCF-7 cells in comparison to control with treatment of CSE at 250, 500, and 1000 *μ*g/ml resulted in a significant decrease of 10%, 23%, and 41%, respectively, in the GSH level. As shown in Figure 4(b), the CSE induced a significant increase in the LPO level. An increase of 15%, 39%, and 97% at 250, 500, and 1000 *μ*g/ml of CSE was found in MCF-7 as compared to control (Figure 4(b)). Further, we measured intercellular ROS production in MCF-7 cells exposed to 0, 250, 500, and 1000 *μ*g/ml of CSE for 24 h. The ROS production was analyzed by two methods (Figure 5): first, the cellular oxidation of DCF-DA, which oxidized to green fluorescent by intracellular ROS under a fluorescence microscope (Figure 5(a)), and quantitative ROS generation measuring the fluorescence of the cell-induced corn silk extract in MCF-7 cells (Figure 5(b)). As shown in Figure 5(a), the green fluorescence intensity clearly indicates the ROS generation induced by corn silk extract. The graph of Figure 5(b) also exhibited a significant increase of 22%, 74%, and 124% at 250, 500, and 1000 *μ*g/ml of CSE, respectively, as compared to control. The previous reports suggest that the natural product endorsed oxidative stress by decreasing the intracellular glutathione level and increasing the lipid peroxidation in the cells [33, 34]. A concentration-dependent decrease in GSH and an increase in LPO levels as found in this study induced by CSE in MCF-7 indicate that oxidative stress plays an important role in the cytotoxicity/cell death. The data obtained in the present study is also supported by previous studies that showed the involvement of oxidative stress in *Nigella sativa* seed oil-induced cell death in human hepatocellular carcinoma cell line [29]. Reactive oxygen species (ROS) produced as a byproduct of cellular metabolism mainly in the mitochondria play a significant role in the cell proliferation, survival, and differentiation [35]. In a normal condition, production and exclusion of ROS are balanced in the cells; however, the stimulation by xenobiotics can cause excessive production of ROS. The excessive production of ROS can lead to oxidative damage, cell cycle arrest, and cellular apoptosis [36, 37]. In this study, we found that CSE increases the production of ROS in a concentration-dependent manner. Therefore, it can be assumed that ROS is involved in the cell death of MCF-7 induced by CSE. These results are also supported by previous studies showing the excessive ROS generation induced by natural products [38–40]. The increased amounts of ROS are known to result in the physiological dysfunction and cell damage [41].

### 3.3. CSE Induced Change in MMP (*ΔΨ*m)

**Figure 6:**
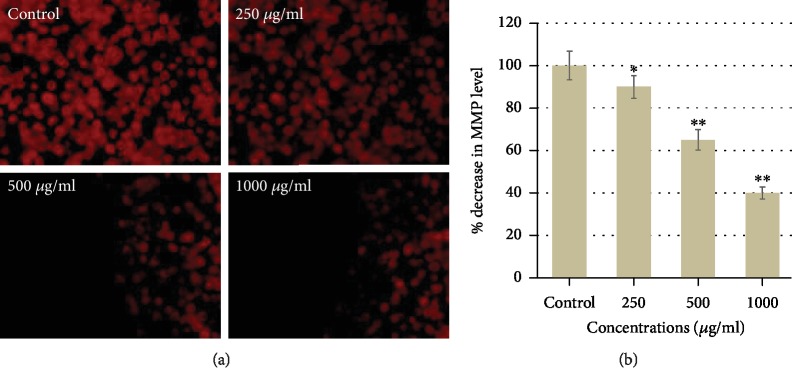
Corn silk extract (CSE) induced loss in MMP. The loss in the MMP level was observed in MCF-7 after the exposure of CSE at 250-1000 *μ*g/ml for 24 h. (a) The fluorescence intensity of Rh123 dye was analyzed under a fluorescence microscope. (b) The graph shows percent loss in the MMP level in MCF-7 induced by corn silk extract. Data are presented as the mean ± SD of three different experiments. ^∗^*p* < 0.05 and ^∗∗^*p* < 0.01 compared to control.

It is known that change in mitochondrial permeability is a vital sign of the cellular apoptosis [42]. Thus, to explore whether CSE can interrupt the *ΔΨ*m, the MCF-7 cells were treated with different concentrations (250, 500, and 1000 *μ*g/ml) of CSE for 24 h. The MMP was observed by staining with cationic fluorescence dye (Rh123). The results found from fluorescence measurements clearly showed a concentration-dependent decrease in the *ΔΨ*m in MCF-7 cells treated with CSE (Figure 6(a)). As shown in Figure 6(b), compared to control, a significant decrease of 10%, 35%, and 60% was found at 250, 500, and 1000 *μ*g/ml of CSE, respectively. As observed in the present investigation, the decrease in *ΔΨ*m is as early incidence of apoptotic cell death in MCF-7 cells. This also indicates the role of the mitochondrial-mediated pathway in apoptosis induced by CSE. Our results are also supported by Lee et al. [43], who have described that corn silk could loss the *ΔΨ*m in prostate cancer (PC-3) cells. Gou et al. [23] have also reported that CSE decreased the *ΔΨ*m in a concentration-dependent manner in LoVo (human colon cancer cell line).

### 3.4. CSE Induced Changes in Apoptotic Marker Genes

**Figure 7:**
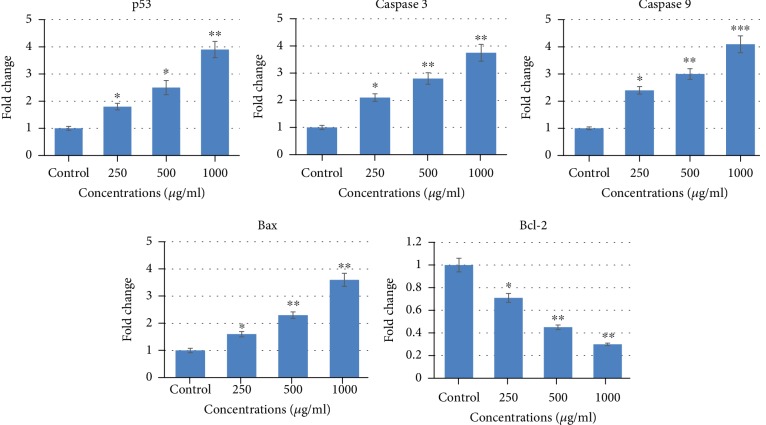
Fold change in the expression of apoptosis-related genes in MCF-7 cells analyzed by real-time PCR (qPCR). MCF-7 cells were exposed to 250-1000 *μ*g/ml of corn silk extract (CSE) for 24 h. The results are presented as the mean ± SD of three different experiments. ^∗^*p* < 0.05, ^∗∗^*p* < 0.01, and ^∗∗∗^*p* < 0.001 compared to control.

As shown in Figure 7, CSE upregulated the mRNA expression of p53, caspase-3, caspase-9, and Bax and downregulated the Bcl-2, which lead to the apoptosis. A concentration-dependent significant increase of 1.8-, 2.5-, and 3.8-fold in the mRNA expression of p53; 2.1-, 2.8-, and 3.75-fold in the mRNA expression of caspase-3; 2.4-, 3-, and 4.1-fold in the mRNA expression of caspase-9; and 1.6-, 2.3-, and 3.6-fold in the mRNA expression of Bax at 250, 500, and 1000 *μ*g/ml of CSE, respectively, was observed. However, a 0.3-, 0.55-, and 0.7-fold decrease in mRNA expression of Bcl-2 at 250, 500, and 1000 *μ*g/ml of CSE, respectively, was observed in MCF-7 cells treated with CSE. The real-time PCR (RT-PCR^q^) data are presented as the expression of apoptotic marker genes (Figure 7). By real-time PCR analysis, we have exhibited that the initiation of the apoptosis pathway induced by CSE exposure to MCF-7 cells was facilitated by disturbing the Bax and Bcl-2 levels. Bax, the proapoptotic gene, has been reported to be upregulated in p53-mediated apoptosis in many systems [44]. Consequently, the increased level of p53 in MCF-7 cells induced by CSE in this study showed that p53 activates the mitochondrial apoptosis. The disturbance in the Bax and Bcl-2 level instigates the dysfunction of the mitochondria, which is encouraging the activation of caspase-3 and caspase-9 [45, 46]. Caspase, a family of proteases, plays a central role in the development of apoptosis. Caspase-9 is involved in the signal transduction cascade, activating caspase-3 that facilitates successive apoptotic signaling. Casapse-3 in the apoptotic pathway plays a critical role during DNA fragmentation, chromatin condensation, and other apoptotic methods [47]. In this study, upregulation in the mRNA expression of caspase-3 and caspase-9 clearly showed that CSE significantly induced apoptosis in MCF-7 cells. A concentration-dependent decrease in the Bcl-2 level observed might have been directed to decrease in MMP (*ΔΨ*m) as shown in this study, following the activation of caspase-3 through the caspase-9 pathway. Our results are also in accordance with the previous report showing that the overexpression of ROS induced by CSE decreases *ΔΨ*m which activates caspase signaling pathways and apoptosis in rat C6 glioma and human colon cancer cell lines [23, 24].

## 4. Conclusions

The present investigation demonstrated that the methanolic extract of corn silk (CSE) induced cytotoxicity in the human breast cancer cell line (MCF-7). There was no cytotoxic effect of CSE observed on normal human mesenchymal (hMSC-TERT4) cells at tested concentrations. The CSE was also found to increase the LPO and ROS production and decrease the GSH level in a dose-dependent manner in MCF-7 cells. The loss in mitochondrial membrane potential indicates the efficacy of CSE against cancer cells. The CSE induced upregulation in proapoptotic marker genes (p53, Bax, caspase-3, and caspase-9) and downregulation in antiapoptotic gene, Bcl-2, which exhibited apoptotic cell death in human breast cancer cells through the ROS-mediated mitochondrial pathway.

## Data Availability

The data used to support the findings of this study are included within the article.
